# Supplementary material for: Stagnant forearc mantle wedge inferred from mapping of shear-wave anisotropy using S-net seafloor seismometers
Source: Nat Commun. 2020 Nov 10;11:5676. doi: 10.1038/s41467-020-19541-y (PMC7655809; doi:10.1038/s41467-020-19541-y)
Supplement: Supplementary file 1 — Supplementary information [file 41467_2020_19541_MOESM1_ESM.pdf]

Supplementary Information

# Stagnant forearc mantle wedge inferred from mapping of shear-wave anisotropy using S-net seafloor seismometers

**Naoki Uchida[1], Junichi Nakajima[2], Kelin Wang[3], Ryota Takagi[1], Keisuke Yoshida[1], Takashi Nakayama[1], Ryota Hino[1], Tomomi Okada[1] and Youichi Asano[4]**

[1]Graduate School of Science, Tohoku University, 6-6, Aramaki-aza-aoba, Aoba-ku, Sendai 980-8578, Japan.

[2] Department of Earth and Planetary Sciences, School of Science, Tokyo Institute of Technology, 2-12-1, Ookayama, Meguro-ku, Tokyo 152-8551, Japan.

[3] Pacific Geoscience Centre, Geological Survey of Canada, 9860 West Saanich Road, Sidney, British Columbia V8L 4B2, Canada.

[4] National Research Institute for Earth Science and Disaster Resilience, 3-1 Tennodai, Tsukuba, Ibaraki 305-0006, Japan.

## Introduction

This supporting material contains back-azimuth and incident angle distribution for 16 stations (Supplementary Figure 1) and comparison of depths by Japan Metrological Agency (JMA) and F-net with S-net based S-P times for earthquakes immediately below stations (Supplementary Figure 2). The table of splitting parameters estimated for each station is provided as separate Supplementary Data1 and 2.

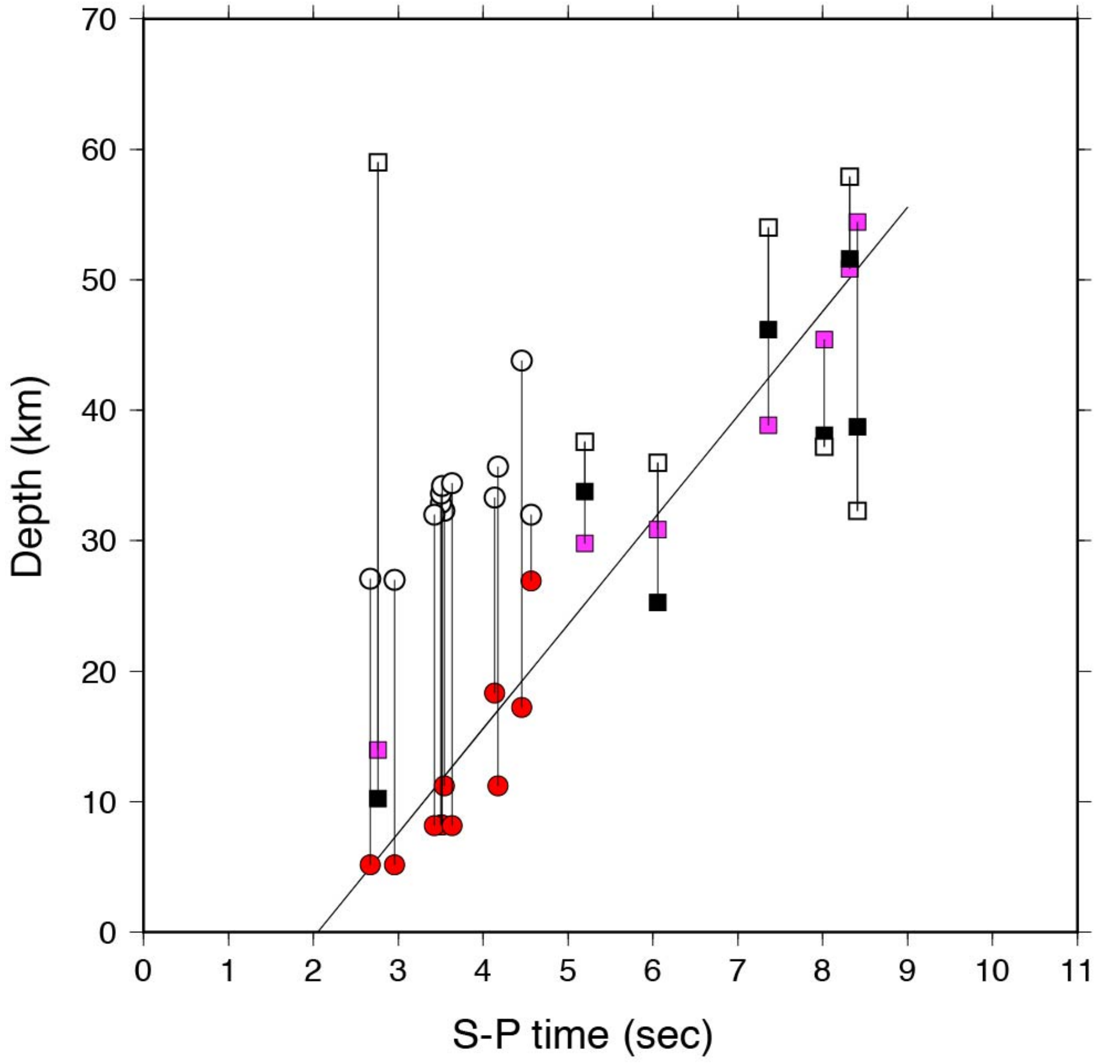


Supplementary Figure 1. The relationship between S-P time observed at S-net stations and focal depth in F-net (red circles and pink squares) and JMA (white circles and squares) catalogues. The black bar connects the same earthquake. The earthquakes shallower than the subduction interface or 35 km, whichever is shallower based on F-net data, are shown with circles, and interplate earthquakes based mainly on focal mechanisms are shown with squares. All earthquakes are with incident angle of 5° or smaller according to JMA's hypocenters. For interplate earthquakes, the depth of plate boundary at JMA's epicenter based on the models of Nakajima and Hasegawa (2006)[1] (Tohoku) and Kita et al., 2010[2] (Hokkaido) are shown with black squares. The thin black line show depth (km) = 2.0 + 8*(S-P time - 2.3)] which assumes 2.0 km sediment layer with very low S-wave speed that produces 2.3 second S-P time. The existence of the sediment layer is well known in this area[3]. The F-net depth and the depth based on plate boundary are near the thin line.

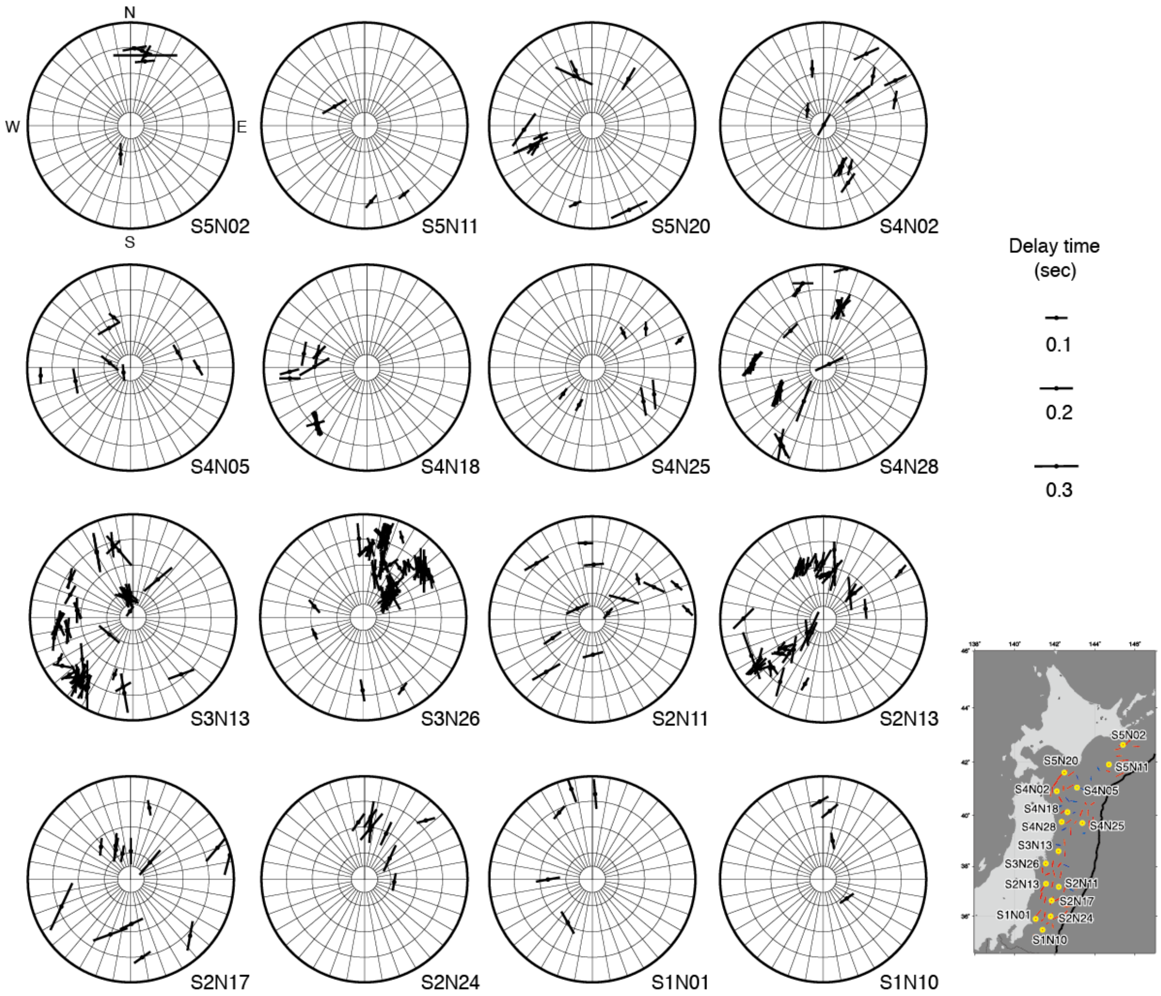


Supplementary Figure 2. The azimuth and incident angle stereogram of the fast directions and delay times for stations shown in bottom right. The incident angles is 0° at the center of the circle and 40° at the edge of the circle. The direction of a bar indicates the fast axis and its length is scaled to the delay time.

Supplementary References

1. Nakajima, J., and A. Hasegawa, Anomalous low-velocity zone and linear alignment of seismicity along it in the subducted Pacific slab beneath Kanto, Japan: Reactivation of subducted fracture zone?, Geophys. Res. Lett., 33, L16309, doi: 10.1029/2006GL026773 (2006).

2. Kita, S., T. Okada, A. Hasegawa, J. Nakajima, and T. Matsuzawa, Anomalous deepening of a seismic belt in the upper-plane of the double seismic zone in the Pacific slab beneath the Hokkaido corner: Possible evidence for thermal shielding caused by subducted forearc crust materials, Earth Planet. Science Lett., 290, 415-426 (2010).

3. Yamamoto, Y. et al. Three-dimensional seismic velocity structure around the focal area of the 1978 Miyagi-Oki earthquake. Geophys. Res. Lett. 33, doi:10.1029/2005GL025619 (2006).
