# Supplementary material for: Stagnant forearc mantle wedge inferred from mapping of shear-wave anisotropy using S-net seafloor seismometers
Source: Nat Commun. 2020 Nov 10;11:5676. doi: 10.1038/s41467-020-19541-y (PMC7655809; doi:10.1038/s41467-020-19541-y)
Supplement: Supplementary file 3 — Description of Additional Supplementary Files [file 41467_2020_19541_MOESM3_ESM.pdf]

Description of Additional Supplementary Files

File name: Supplementary Data 1

Description: Average fast direction and delay time for each station for interplate earthquakes. For the standard deviation (STD) of the fast direction, we used the circular standard deviation that utilizes the directions and lengths of the summed unit vectors [Mardia and Jupp, 2009]. If the number of earthquakes is smaller than 4, the standard deviation (representing uncertainties) is not provided for the delay time or fast direction.

File name: Supplementary Data 2

Description: Average fast direction and delay time for each station for upper-plate (depth < 35km) earthquakes. See Supplementary data 1 for the explanation of the standard deviations (STD).
